# Outbreak of carbapenem-resistant enterobacteria in a thoracic-oncology unit through clonal and plasmid-mediated transmission of the *bla*_OXA-48_ gene in Southern France

**DOI:** 10.3389/fcimb.2022.1048516

**Published:** 2022-12-07

**Authors:** Linda Hadjadj, Nadim Cassir, Nadia Saïdani, Clémence Hoffman, Philippe Brouqui, Philippe Astoul, Jean-Marc Rolain, Sophie Alexandra Baron

**Affiliations:** ^1^ Aix Marseille Univ, Institut de recherche pour le développement (IRD), Assistance Publique-Hôpitaux de Marseille (APHM), Microbes, Evolution, Phylogénie et Infection (MEPHI), Faculté de Médecine et de Pharmacie, Marseille, France; ^2^ Institut hospitalo-universitaire (IHU) Méditerranée Infection, Marseille, France; ^3^ Service de Maladies infectieuses et tropicales, Centre Hospitalier de Quimper, Quimper, France; ^4^ Department of Thoracic Oncology, Pleural Diseases, and Interventional Pulmonology, North University Hospital, Marseille, France; ^5^ Aix-Marseille University, Marseille, France

**Keywords:** whole genome sequencing, carbapenemase, *bla*_OXA-48_ gene, IncL/M plasmid, fecal microbiota transplantation

## Abstract

**Background:**

Carbapenemase-producing *Enterobacteriaceae* (CPE) represent an increasing threat to public health, especially in hospitals.

**Objectives:**

To investigate an outbreak of CPE in a thoracic-oncology unit by using whole genome sequencing (WGS) and to describe the control measures taken to limit the epidemic, including fecal microbiota transplantation (FMT).

**Methods:**

A retrospective study between December 2016 and October 2017 was performed to investigate an outbreak of CPE in a thoracic-oncology unit at the North Hospital in Marseille, France. The isolates were identified, and antimicrobial susceptibility tests were performed. All CPE were sequenced using MiSeq and/or MinIon technologies. Nucleotide variations between plasmids and similarity within the same species were investigated. The origin of this outbreak, its spread, and the decolonization of patients in the ward were also studied.

**Results:**

Four *Citrobacter freundii*, one *Enterobacter cloacae* and four *E. hormaechei* OXA-48 carbapenemase producers were isolated in eight patients hospitalized the same year in a thoracic-oncology ward. The *bla*_OXA-48_ gene was present in a Tn*1999.2* transposon located in IncL/M plasmids, with single nucleotide variants (SNV) ranging from 0 to 5. All *C. freundii* strains belonged to the same ST22 and had more than 99.6% similarity between them. Two strains of *E. hormaechei* ST1007 were almost identical at 99.98%, while the others belonged to a different ST (ST98, ST114, ST133). No single source was identified. FMT resulted in decolonization in 4/6 patients.

**Conclusions:**

WGS demonstrated the dissemination of the *bla*_OXA-48_ gene by both clonal (*C. freundii* ST22 and *E. hormaechei* ST1007) and plasmid spread (pOXA-48 IncL/M). The origin of this outbreak appeared to be both external and internal to the ward. This evidence of cross-infection supports the urgent need for the implementation of infection control measures to prevent CPE dissemination.

## Introduction

Carbapenems are broad-spectrum antibiotics that play a major role in the treatment of severe infections caused by Gram-negative bacteria. The global spread of carbapenem-resistant *Enterobacteriaceae* is becoming a public health issue (Jamal et al., 2020). The rise of carbapenem resistance in *Enterobacteriaceae* is mainly due to the acquisition of carbapenem-hydrolyzing enzymes (carbapenemases) (Tilahun et al., 2021). Genes encoding carbapenemases may be incorporated into the bacterial chromosome, but are mostly located on mobile elements, such as plasmids or transposons that are transferable between bacterial strains and species (San Millan, 2018). Hence, clinical outbreaks are usually complex, involving various factors of gene propagation by clones, plasmids, or transposons (Brehony et al., 2019).

Carbapenemase type OXA-48 first appeared in the mid-2000s in Turkey and has since been found in many European countries and worldwide (Hidalgo et al., 2019). In France, it is the most common enzyme among carbapenemase-producing *Enterobacteriaceae* (CPE) (Emeraud et al., 2020). The *bla*_OXA-48_ gene is thought to originate from the chromosome of environmental *Shewanella* strains (Tacão et al., 2018). Its rapid dissemination between species is due to its nesting in a transposon (Tn*1999*) that is carried primarily by IncL/M type plasmids (Shankar et al., 2020).

Controlling outbreaks in hospital wards is necessary to limit the spread of multidrug-resistant bacteria. The colonization of patients by CPE can interfere with proper care. CPE colonization can also have an impact on the initiation of chemotherapy in cancer patients, as it has been associated with a lower survival rate in patients undergoing induction chemotherapy (Ballo et al., 2019). Thus, a strategy to restore a healthy gut microbiota and to eliminate the CPE reservoir such as fecal microbiota transplantation (FMT) has been implemented. FMT is a validated therapy that is highly effective against recurrent *Clostridium difficile* infections (Yoon et al., 2019). In addition, recent studies have shown that FMT was an efficient strategy for sustained CPE eradication. (Decraene et al., 2018; Saïdani et al., 2019). However, there could be other reservoirs of CPE. For instance, the survival of CPE on surfaces also allows the resumption of epidemics months after the initial case (Mateos et al., 2020).

During nosocomial outbreaks, the transmission of pathogens in a ward can be studied by whole genome sequencing of the bacteria of interest, a highly discriminatory typing technique. The genetic relationship between strains, as well as the presence of antibiotic resistance genes and their genetic support, can be determined very accurately and completely (Jamal et al., 2020). However, short-read sequencing (Illumina technology) is not always suitable for locating antibiotic resistance genes on plasmids, whereas long-read sequencing (Nanopore technology) can solve this problem. Therefore, a hybrid assembly of both highly accurate Illumina data and Nanopore data allow the reconstitution of plasmids and their in-depth analysis (George et al., 2017). In our study, these technologies were used to investigate the source and mode of dissemination of the *bla*_OXA-48_ gene in different *Enterobacteriaceae* during an outbreak in an adult thoracic-oncology unit in Marseille, France. The management of this epidemic by clinicians and the decolonization of patients are also described.

## Methods

### Study design and bacterial strains

**Figure 1:**
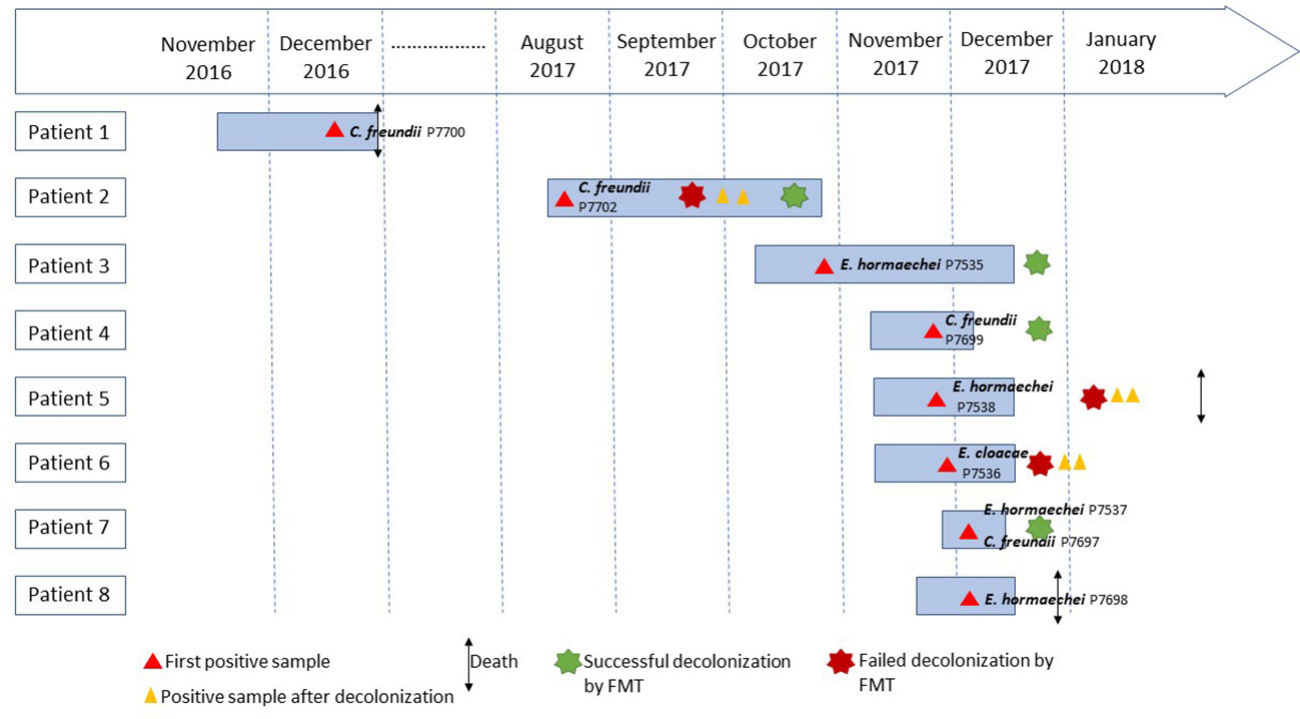
Timeline of patients with carbapenemase-producing *Enterobacteriaceae*.

This retrospective study was conducted to investigate an outbreak of CPE in six patients that occurred between October and December 2017 in a thoracic-oncology center at the North hospital of Marseille, France. To perform this investigation, we also extracted medical records from two other patients that had been positive for CPE in the same ward, one in December 2016 and one between August and October 2017 (**Figure 1**; **Table 1**). From these eight patients, nine strains were isolated, including eight from rectal swabs and one from a urine sample. Patient 2 carried two different CPE isolates. An epidemiological investigation was conducted to understand the source of this outbreak. The mode of dissemination of the *bla*_OXA-48_ gene and the relatedness between isolates were studied using whole-genome sequencing.

**Table 1:** Clinical information on patients with OXA-48 carbapenemase.

Patient	CPE	Sex	Age	Main diagnosis	Isolation date	Date of admission	Date of dischearge	Hospitalization days	Antibiotic susceptibility testing		Colonization	Decolonization method	CPE decolonization
									Susceptible phenotype	Resistant phenotype			
Patient 1	*C. freundii* P7700	M	59	Lung adenocarcinoma	18/12/2016	21/11/2016	30/12/2016	39	FOF, AK, FEP, ERT (1), IPM (1)	AMX, AMC, CRO, TPZ, CIP, DO, GEN, SXT	U	Dead before FMT	/
Patient 2	*C. freundii* P7702	F	64	Recurrent hemoptysis	17/08/2017	14/08/2017	27/10/2017	74	FOF, AK, FEP, ERT (0.5), IPM (0.5)	AMX, AMC, CRO, TPZ, CIP, DO, GEN, SXT	R/U	FMT by gastric way (2 times)	Failure of the first attempt but success of the second
Patient 3	*E. hormaechei* P7535	F	73	Lung adenocarcinoma	27/10/2017	10/10/2017	19/12/2017	40	FOF, AK, GEN, FEP, DO, SXT	AMX, AMC, CRO, TPZ, CIP, ERT (2), IPM (2)	R	FMT by gastric way	Success
Patient 4	*C. freundii* P7699	F	56	Urothelial carcinoma	22/11/2017	12/11/2017	02/12/2017	20	FOF, AK, FEP, ERT (1), IPM (1)	AMX, AMC, CRO, TPZ, CIP, DO, GEN, SXT	R	FMT by gastric way	Success
Patient 5	*E. hormaechei* P7538	M	71	Laryngeal carcinoma	22/11/2017	11/11/2017	15/12/2017	34	FOF, AK, ERT (1), IPM (1)	AMX, AMC, CRO, TPZ, CIP, FEP, DO, SXT, GEN	R/C	FMT by gastric way	Failure
Patient 6	*E. cloacae* P7536	F	28	Pleurisy on gastropleural fistula	30/11/2017	13/11/2017	17/12/2017	34	FOF, AK	AMX, AMC, CRO, TPZ, CIP, FEP, DO, SXT, GEN, ERT (4), IPM (2)	R	FMT by rectal way	Failure
Patient 7	*C. freundii* P7697	F	57	Lung adenocarcinoma	7/12/2017	27/11/2017	13/12/2017	16	FOF, AK, FEP, ERT (1), IPM (1)	AMX, AMC, CRO, TPZ, CIP, DO, GEN, SXT	R/U	FMT by gastric way	Success
	*E. hormaechei* P7537								FOF, AK, FEP, DO, SXT, GEN	AMX, AMC, CRO, TPZ, CIP, ERT (2), IPM (2)			
Patient 8	*E. hormaechei* P7698	M	63	Small cell lung carcinoma	7/12/2017	18/11/2017	16/12/2017	28	FOF, AK	AMX, AMC, CRO, TPZ, CIP, FEP, DO, SXT, GEN, ERT (>32), IPM (6)	R/U/C	Dead before FMT	/

CPE, Carbapenemase-producing Enterobacteriaceae; R, Rectal; U, Urinary; C, Cutaneous; FMT, Fecal Microbiota Transplantation; FOF, fosfomycin; AK, amikacin; AMX, amoxicillin; AMC, amoxicillin-clavulanic acid; CRO, ceftriaxone; TPZ, piperacillin-tazobactam; CIP, ciprofloxacin; FEP, cefepime; DO, doxycycline; SXT, trimethoprim-sulfamethoxazole; GEN, gentamicin; ERT, ertapenem; IPM, imipenem; (MIC en µg/mL).

### Decolonization by fecal microbiota transplantation

As previously noted, FMT was performed in six patients (Patients 2 to 7) (Saïdani et al., 2019). Briefly, patients who underwent FMT were previously sampled for various colonization sites (urine, pharynx, nasopharynx, and rectum) and additional potential sites (gastrostomy, skin, wounds, etc.). This mapping of CPE-colonized sites was established over three consecutive days prior to the FMT protocol. Eight days before FMT, a 3-day nasopharyngeal decolonization (in case of nasopharyngeal carriage) was performed using 0.12% chlorhexidine gluconate as local treatment of the mouth (gargling) and nasal cavities (swab applications). Five days before FMT, patients received an initial bowel lavage (until stools became watery and clear). An oral non-absorbable combination of antibiotics comprising colistin 6 MIU every 6h and amikacin 200 mg every 6h was then administered, replaced by other antibiotics in case of resistance, according to isolated CPE antibiotic susceptibility. One day prior to FMT, the patient received a second bowel lavage (until stools became watery and clear) and was given a proton-pump inhibitor (pantoprazole 40 mg twice a day for 48h).

Fecal microbiota transplantation was only considered for CPE-colonized patients for whom rehabilitation, surgery or chemotherapies were indicated and were likely to be delayed, based on the argument that CPE carriage would lead to a consequent loss of opportunity.

### Phenotypic and molecular analyses

All strains were isolated on chromID^®^ CARBA SMART agar (bioMérieux, Marcy-l’Etoile, France), except for P7700 on Columbia agar with 5% sheep blood (bioMérieux). Isolates were identified by matrix-assisted laser desorption and ionization time-of-flight mass spectrometry (MALDI-TOF) (Microflex, Brüker Daltonics, Bremen, Germany) (Seng et al., 2009). Antimicrobial susceptibility testing (AST) was performed using the disc diffusion method on a panel of 13 antibiotics (i2a, Montpellier, France): amoxicillin, amoxicillin-clavulanic acid, cefepime, ceftriaxone, piperacillin-tazobactam, ertapenem, imipenem, fosfomycin, doxycycline, trimethoprim-sulfamethoxazole, amikacin, gentamicin and ciprofloxacin, according to EUCAST recommendations (version 3.1). The minimum inhibitory concentration (MIC) of ertapenem and imipenem was determined by the E-test method (bioMérieux, Marcy l’Etoile, France). The β-CARBA test (Bio-Rad, Hercules, CA, USA) was used to detect carbapenemase production.

Real-time PCR (RT-PCR) was performed in all strains to confirm the presence of genes encoding carbapenem hydrolyzing enzymes (*bla*_NDM_, *bla*_KPC_, *bla*_OXA-48_, *bla*_VIM_) using the CFX96 Touch Real-Time PCR Detection System (Bio-Rad, Hercules, CA, USA). Targeted genes were detected using specific primers and Taqman probes (Yousfi et al., 2019).

### Genomic analyses

Total genomic DNA (gDNA) was extracted using a EZ1 DNA kit and the BioRobot EZ1 (Qiagen, Courtaboeuf, France) according to the manufacturer’s instructions. Then, the gDNA was quantified by a Qubit assay (Life Technologies, Carlsbad, CA, USA) and its quality was controlled by Bioanalyser systems (Agilent, Santa Clara, CA, USA). All CPEs were sequenced in 2x250 bp paired-end reads in MiSeq (Illumina Inc., San Diego, CA, USA), of which two *Citrobacter freundii* strains (P7697, P7699) and two *Enterobacter* sp. isolates (P7536, P7538) were also sequenced using MinIon technology (Oxford Nanopore Technologies Inc., United Kingdom). The runs performed by the MinION technology were done using Ligation Sequencing Kit (Oxford Nanopore Technologies Inc., United Kingdom) and the libraries were loaded on a flow cell R9.4.1. Spades software (Bankevich et al., 2012) was used to assemble the Illumina generated sequencing data and also for the assembly of the mixed Nanopore-Illumina data. Genome annotation, antibiotic resistance gene, and plasmid screening were performed with RAST (Aziz et al., 2008), Resfinder (Bortolaia et al., 2020) and PlasmidFinder (Carattoli et al., 2014), respectively. The CPE sequence type (ST) was performed *in silico* using multilocus sequence typing (MLST) analysis on the Center for Genomic Epidemiology website (https://www.genomicepidemiology.org/).

The genetic environment of the *bla*_OXA-48_ genes was reconstructed by comparing the sequences of the genes surrounding this gene to the NCBI database using the blastX parameter. The complete plasmid sequences of strains P7697, P7699, P7536 and P7538 carrying *bla*_OXA-48_ genes were compared with the reference plasmid CP027039.1 using CGViewServer software (Stothard et al., 2017). Variant calling on the snippy tool version 4.6.0 was used to detect single nucleotides variants (SNV), both between *bla*_OXA-48_ plasmids and between strains of the same species. Pangenome analysis was performed with Roary (Page et al., 2015) and visualized using Phandango software (Hadfield et al., 2018). The percent similarity of the *C. freundii* strains and *Enterobacter* sp. strains was calculated by pairwise comparison of their average nucleotide identity based on Blast (ANIb) and using JSpecies (Richter et al., 2016).

Genomes of P7535, P7536, P7537, P7538, P7697, P7698, P7699, P7700 and P7702 strains have been submitted to GenBank under accession numbers JAGDEG000000000, CP071788- CP071792, JAGDEH000000000, CP071830-CP071833, CP071834-CP071838, JAGDEI000000000, CP071907-CP071913, JAGDEJ000000000, and JAGDEK000000000, respectively (**Table 2**).

**Table 2:** Genomic analysis of *C. freundii* and *Enterobacter* sp. strains.

Isolate	ST	Plasmid replicate	ARGs	Genbank accession number
*E. hormaechei* P7535	1007	Chr- IncFIB	*bla*_ACT-15_, *bla*_LAP-2_, qnrS1, fosA	JAGDEG000000000
		**IncL/M**	***bla*_OXA-48_**	
*E. cloacae* P7536	98	Chr	*bla*_ACT-16_, aadA1, sul1, catA1, fosA	CP071792
		IncFII	*bla*_OXA-9_, *bla*_TEM-1A_, aac(6’)Ib-cr, aadA1	CP071790
		IncHI2	*bla*_OXA-1_, *bla*_TEM-1A_, *bla*_CTX-M-15_, qnrB1, aac(3)-IIa, aac(6’)Ib-cr, aph(3’’)-Ib, aph(6)-Id, aadA1, catA1, catB3, sul2, dfrA14, tet(A)	CP071791
		IncA	aac(6’)Ib-cr, aadA1, catB2, sul1	CP071789
		**IncL/M**	***bla*_OXA-48_**	CP071788
E. hormaechei * P7537	1007	Chr– IncFIB	*bla*_ACT-15_, *bla*_LAP-2_, qnrS1, fosA	JAGDEH000000000
		**IncL/M**	***bla*_OXA-48_**	
*E. hormaechei* P7538	133	Chr	*bla*_ACT-7_, fosA	CP071833
		IncFIB	None	CP071831
		IncHI2	*bla*_OXA-1_, *bla*_TEM-1B_, *bla*_CTX-M-15_, qnrB1, aac(6’)Ib-cr, aph(3’’)-Ib, aph(6)-Id, aadA1, catA1, catB3, sul2, dfrA14, tet(A)	CP071832
		**IncL/M**	***bla*_OXA-48_**	CP071830
*E. hormaechei* P7698	114	Chr- IncFIB	*bla*_OXA-1_, *bla*_TEM-1B_, *bla*_DHA-1_, *bla*_ACT-16_, qnrB2, qnrB4, fosA, mph(A), aac(3)-IId, aac(6’)Ib-cr, aadA1, catB3, sul1, dfrA1, ARR-3, tet(D)	JAGDEI000000000
		**IncL/M**	***bla*_OXA-48_**	
C. freundii* P7697	22	Chr	*bla*_CMY-48_, aadA1, dfrA1	CP071834
		pKPC-CAV1193	*bla*_SHV-12_	CP071838
		IncA	None	CP071837
		Plasmid	*bla*_OXA-1_, mph(A), aac(6’)Ib-cr, catB3, sul1, sul2, ARR-3, tet(D)	CP071836
		Unlocated	*bla*_TEM-1B_, *bla*_OXA-10_, aac(3)-IId, aac(6’)Ib-cr	/
		**IncL/M**	***bla*_OXA-48_**	CP071835
*C. freundii* P7699	22	Chr	*bla*_CMY-48_, aadA1, dfrA1	CP071907
		IncA	aac(6’)Ib-cr, aadA1, catB2, sul1	CP071911
		pKPC-CAV1193	*bla*_SHV-12_	CP071913
		IncHI1A	None	CP071912
		Plasmid	*bla*_OXA-1_, mph(A), aac(6’)-Ib-cr, catB3, sul1, ARR-3	CP071909
		Plasmid	*bla*_TEM-1B_, catA2, sul2, tet(D)	CP071910
		**IncL/M**	***bla*_OXA-48_**	CP071908
*C. freundii* P7700	22	Chr-pKPC-CAV1193- IncA	*bla*_CMY-48_, *bla*_OXA-1_, *bla*_TEM-1B_, *bla*_SHV-11_, aac(3)-IId, aac(6’)Ib-cr, aadA1, mph(A), catB3, catB2, sul1, sul2, dfrA1, ARR-3, tet(D)	JAGDEJ000000000
		**IncL/M**	***bla*_OXA-48_**	
*C. freundii* P7702	22	Chr- pKPC-CAV1193- IncA	*bla*_CMY-48_, *bla*_OXA-1_, *bla*_TEM-1B_, *bla*_SHV-11_, aac(3)-IId, aac(6’)Ib-cr, aadA1, mph(A), catB3, catB2, sul1, sul2, dfrA1, ARR-3, tet(D)	JAGDEK000000000
		**IncL/M**	***bla*_OXA-48_**	

*Strains isolated from the same patient.

Bold means Informations concerning the IncL/M plasmid.

### Ethics

No sampling was performed for research purposes. Phenotypic, molecular and genomic analyses were performed on bacteria isolated for diagnostic as routine care for epidemiological investigation of the outbreak and infection control intervention. According to European General Data Protection Regulation No. 2016/679, the study was registered under N° 2022-28 in the APHM register.

## Results

### Outbreak control and decolonization by FMT

Overall, eight CPE carriers in a thoracic-oncology ward were identified (**Table 1**; **Figure 1**). The detection of CPE in patient 3 led to the implementation of measures to control the transmission of the outbreak according to current French recommendations (Lepelletier et al., 2015). Initially, the patient was placed on contact isolation precautions, associated with enhanced environmental disinfection, and a training for the healthcare staff on reduction of the risk of cross-transmission was done. In addition, screening of 49 contact patients hospitalized in the thoracic-oncology ward was performed. Five (5/49, 10.2%) new secondary cases (patients 4 to 8) were detected in the thoracic-oncology unit (**Table 1**; **Figure 1**). Isolation and prevention policies were also applied to these patients.

Before this outbreak, two patients carried a CPE at different times. The detection of CPE in patient 1 was confirmed after he died of his oncological illness on the ward, one year before the outbreak. Consequently, no isolation measures were taken. Patient 2 was hospitalized two months before the outbreak and was still hospitalized when patient 3 was detected positive. Patient 2 had been hospitalized abroad within 12 months, so he was placed on contact isolation precautions on arrival in the ward and was evaluated for the carriage of CPE according to the recommendations of the French High Committee for Public Health (Lepelletier et al., 2015). Isolation measures were maintained after the finding of *C. freundii* OXA-48 carriage.

In addition to hand hygiene and contact isolation policies, treatment by selective personalized decolonization and FMT was performed in 6/8 patients so that they could receive chemotherapy. Four out of six patients had a successful FMT (patient 2, 3, 4, 7) confirmed by three negative consecutive CPE controls after transplantation. Two successive FMTs were required to decolonize patient 2 (**Table 1**; **Figure 1**).

A 12-month follow-up did not detect additional CPE carriers in this thoracic-oncology ward.

### Isolates and antibiotic susceptibility testing

Four *C. freundii* strains and five *E. cloacae* strains were isolated from eight clinical samples. All CPE were sensitive to fosfomycin and amikacin but resistant to amoxicillin, amoxicillin-clavulanic acid, ceftriaxone, piperacillin-tazobactam, and ciprofloxacin. All strains of *C. freundii* had the same resistance phenotype (doxycycline, gentamicin, trimethoprim-sulfamethoxazole resistant and cefepime sensitive). Concerning the *Enterobacter* strains, two phenotypes were observed. The isolates P7535 and P7537 were sensitive to cefepime, doxycycline, trimethoprim-sulfamethoxazole and gentamicin, while the remaining (P7536, P7538, P7698) were not (**Table 1**).

Ertapenem and imipenem MICs of *C. freundii* strains varied from 0.5 to 1 µg/mL and 0.5 to 1 µg/mL respectively, while ertapenem and imipenem MICs of *Enterobacter* isolates ranged respectively from 1 to >32 µg/mL and 1 to 6µg/mL (**Table 1**). The β-CARBA test was positive for all isolates that carried only the *bla*_OXA-48_ carbapenemase gene.

### Resistome

All strains of *C. freundii* had a median length of 5.2Mb and a median GC of 51.7% while the median length and GC of *Enterobacter* isolates ranged between 4.98 to 5.1Mb and between 55 to 55.1%, respectively. RT-PCR and genome analysis confirmed that OXA-48 carbapenemase was produced by all strains. Resistome analysis showed the presence of genes encoding resistance to different families of antibiotics such as β-lactams, quinolones, aminoglycosides, sulfonamides, cyclins, and chloramphenicol (**Table 2**).

**Figure 2:**
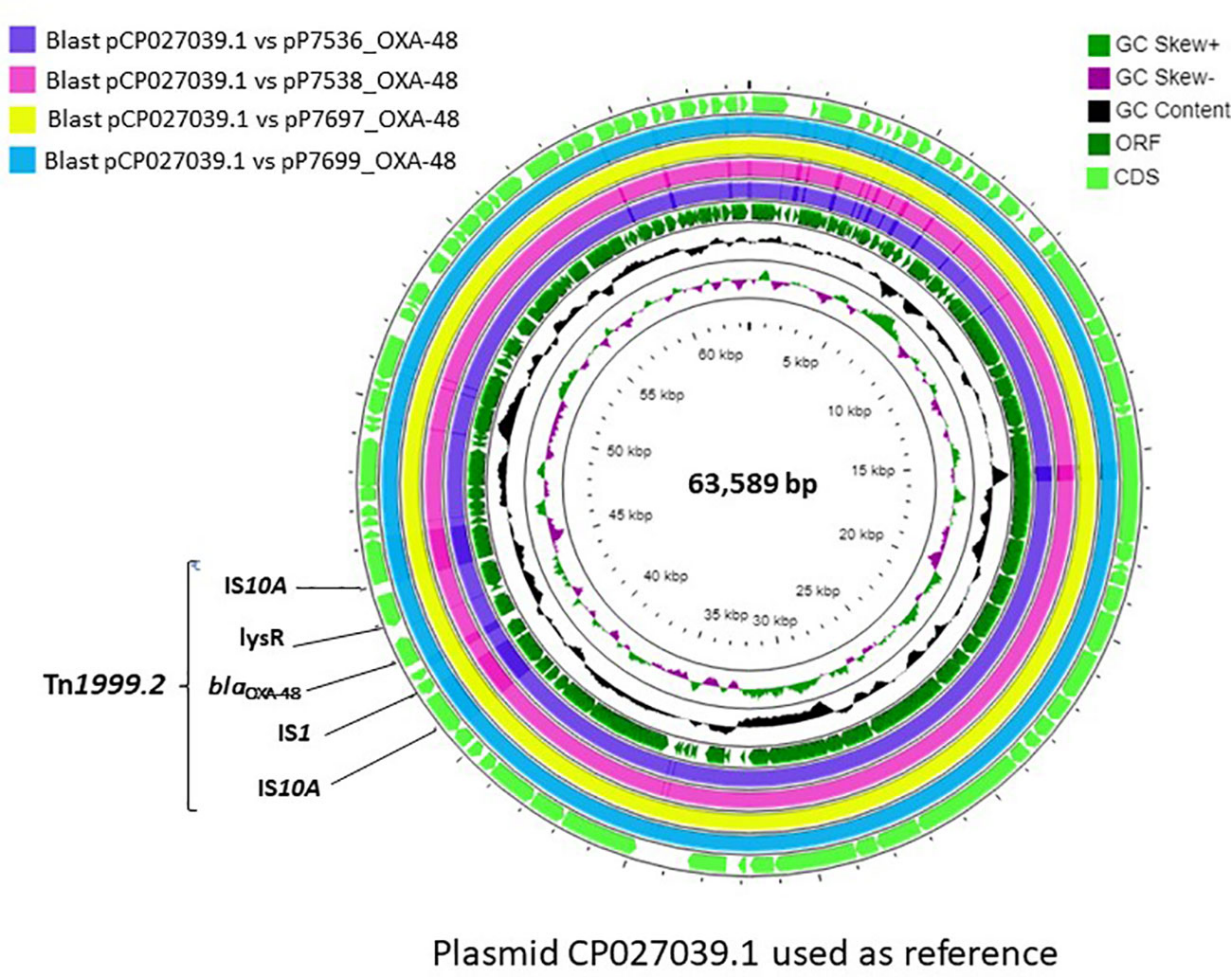
Genetic environment of the *bla*_OXA-48_ gene and circular representation of the complete OXA-48 plasmids.

The gene encoding the OXA-48 enzyme was found in a 63kb IncL/M plasmid, whereas the other antibiotic resistance genes were present on other plasmid types or in the chromosome (**Table 2**). In all CPE, the *bla*_OXA-48_ gene was in a Tn*1999.2* transposon (IS10A-lysR-*bla*_OXA-48_-IS1-IS10A) (**Figure 2**). Comparison of the complete OXA-48 plasmids with all CPE showed the quasi-similarity of the outbreak plasmids, with presence of 0 to 5 SNV.

**Figure 3:**
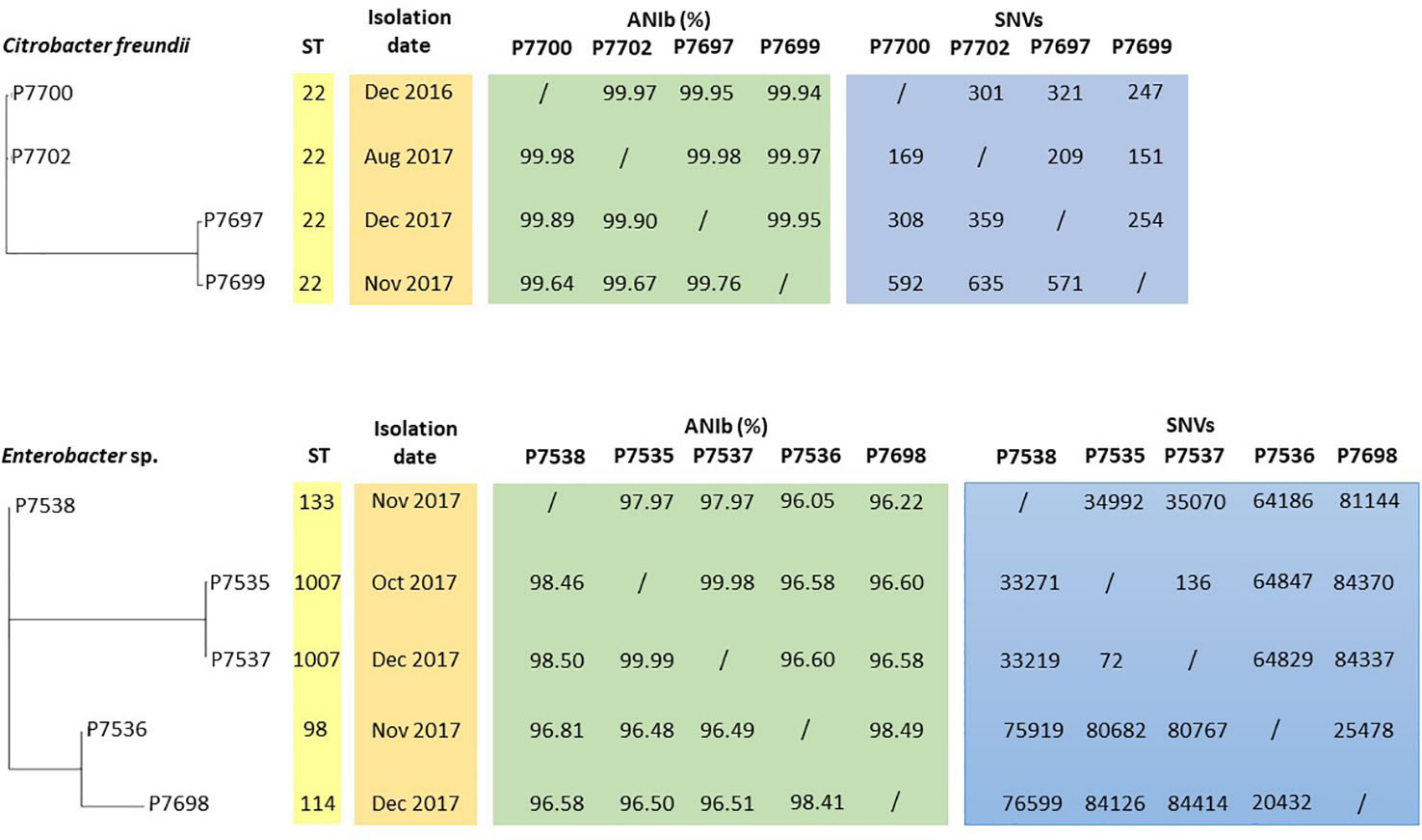
Phylogenetic tree based on the pangenome of *C. freundii* and *Enterobacter* sp. strains associated with ST, isolation date, ANIb and SNV data.

Annotation of genomes on NCBI allowed reclassification of P7535, P7537, P7538, P7698 isolates as *Enterobacter hormaechei* strains (**Table 2**). According to MLST analysis, all *C. freundii* strains belonged to the same ST22 sequence type, while *Enterobacter* isolates had different ST, except P7535 and P7537, which were ST1007 (**Figure 3**; **Table 2**). *E. hormaechei* strains P7535 and P7537 had 99.98% ANIb similarity, while the similarity between the other *Enterobacter* isolates varied from 96.1 to 98.5%. *The C. freundii* strains also shared high similarity rates, from 99.64 to 99.98%. Pangenome and SNV analysis confirmed the same origin for *E. hormaechei* isolates P7535 and P7537, whereas the distance of *C. freundii* isolates indicated that they were related but appear to have diverged over time (**Figure 3**).

## Discussion

The emergence and spread of CPE in the hospital setting are a major concern for clinicians. In this study, among the CPE, we isolated *C. freundii* and *E. hormaechei*, which belong to the *E. cloacae* complex and are identified as *E. cloacae* by most biochemical methods (Mateos et al., 2020). The presence of *C. freundii* producing OXA-48 carbapenemase is emerging in clinical settings where recent reports from Spain warned of an increase in its incidence. In Germany, the ST22 clone of OXA-48 carbapenemase producing *C. freundii* is increasingly noted in the hospital setting (Villa et al., 2017; Lalaoui et al., 2019; Yao et al., 2021). Recently in the north of France, an outbreak of OXA-48 carbapenemase -producing *Enterobacteriaceae* species, including *Enterobacter* sp. and *C. freundii* ST22, has been reported in a haematological ward (Jolivet et al., 2021).

IncL/M plasmids are known to be major carriers of the *bla*_OXA-48_ gene, which is very commonly inserted into a Tn1999-type transposon (Giani et al., 2012). Tn*1999.2*, present in all of our strains, has been found in different species of *Enterobacteriaceae* in Europe (Hidalgo et al., 2019). In our case, the same IncL/M OXA-48 plasmid was propagated among the different strains of *C. freundii* and *Enterobacter* sp. The horizontal transfer of conjugative plasmid is a key factor in the spread of antibiotic resistance genes in different clones and species of *Enterobacteriaceae*. It allows the evolution of resistance in certain bacterial clones due to an efficient bacterial-plasmid association (San Millan, 2018). This phenomenon was observed in a Spanish hospital in which a *Klebsiella pneumoniae* ST11 clone carrying OXA-48 carbapenemase was identified in 44 patients (Branas et al., 2015). In our case, similar clones of *C. freundii* circulated in the ward for one year, and the same strain of *E. hormaechei* spread between patients 3 and 7.

In our study, all patients colonized by CPE were hospitalized in the thoracic-oncology ward. Rectal or urinary colonization with CPE and prolonged hospital stays are risk factors for infections by these pathogens (Mateos et al., 2020). Spontaneous decolonization of intestinal carriage in patients colonized with CPE is a common but slow event (Zimmerman et al., 2013). FMT is a validated procedure that allows reduction of the time of colonization and thus accelerates medical management for patients (Saïdani et al., 2019). In our case, CPE colonization of cancer patients led to the postponement of chemotherapy treatment due to a higher risk of CPE infections in immunocompromised patients. Therefore, the benefits of FMT was to reduce the delay in the management of these patients, thus reducing their loss of opportunity (Saïdani et al., 2019).

The source of initial contamination of patient 1, at the origin of this outbreak, has not been identified to date, but an environmental track is probable and should not be neglected. The hospital environment and especially inanimate surfaces have often been identified as a reservoir for multidrug-resistant bacterial outbreaks (Decraene et al., 2018). Many Gram-negative species can survive on these surfaces for as long as several months (Pantel et al., 2016). One reason for the persistent transmission of OXA-48 *C. freundii* in a hematology department was the presence of contaminated toilets which constituted a potential reservoir (Jolivet et al., 2021). In our case, the same clone of *C. freundii* was present in four different patients, hospitalized in different rooms, at different times, which is worrying. Therefore, the origins of this clone seem to be both external and internal to the unit. On the one hand, it could be due to the persistence of CPE in the department after the first patient died in 2016. Before the outbreak, patients were not systematically screened on this ward, unless they came from another hospital. On the other hand, the outbreak could have started with patient 2, despite the implementation of isolation precaution procedures. In particular, hand hygiene, the use of gloves, protective clothing and single-use sterile consumables, the excreta management as well as reinforced disinfection of the environment and technical areas are essential in the reduction of the cross-transmission risk (Lepelletier et al., 2015). Systematic screening and isolation of patients upon entry to the unit may be an efficient way to solve this issue and to allow the best possible control of CPE dissemination, although these measures are cumbersome to implement. (Pantel et al., 2014). These procedures are even more important in wards caring for immunocompromised patients (Nicolas-Chanoine et al., 2019). The spread of the same OXA-48 plasmid through different bacterial species and clones has been established. Nevertheless, the measures of control, implemented in accordance with the recommendations of the French High Committee for Public Health after the detection of this outbreak, together with the implementation of an FMT procedure for CPE carriers, promptly controlled this outbreak (Lepelletier et al., 2015).

## Conclusion

Whole genome sequencing demonstrated several modes of transmission of OXA-48 carbapenemase through specific clones, plasmids, and transposons. The emergence and spread of CPE over a period of one year in our hospital is a worrisome development. This study highlights the necessity of investigating the source of contamination and controlling it as soon as possible to avoid the persistence of risky clones within a unit. Despite the precautions taken in the thoracic-oncology ward, this outbreak occurred, demonstrating the difficulty in prevention and control. Decolonization by FMT is an effective procedure that allowed a rapid resumption of oncological treatments. Nevertheless, it is necessary to remain vigilant and to continue epidemiological surveillance, especially when new patients are admitted to a unit, particularly in wards with immunosuppressed patients.

## Data availability statement

The datasets presented in this study can be found in online repositories. The names of the repository/repositories and accession number(s) can be found below: https://www.ncbi.nlm.nih.gov/genbank/, JAGDEG000000000, https://www.ncbi.nlm.nih.gov/genbank/, CP071788-CP071792, https://www.ncbi.nlm.nih.gov/genbank/, JAGDEH000000000, https://www.ncbi.nlm.nih.gov/genbank/, CP071830-CP071833, https://www.ncbi.nlm.nih.gov/genbank/, CP071834-CP071838, https://www.ncbi.nlm.nih.gov/genbank/, JAGDEI000000000, https://www.ncbi.nlm.nih.gov/genbank/, CP071907-CP071913, https://www.ncbi.nlm.nih.gov/genbank/, JAGDEJ000000000, https://www.ncbi.nlm.nih.gov/genbank/, JAGDEK000000000.

## Ethics statement

No sampling was performed for research purposes. Phenotypic, molecular and genomic analyses were performed on bacteria isolated for diagnostic as routine care for epidemiological investigation of the outbreak and infection control intervention. According to European General Data Protection Regulation No. 2016/679, the study was registered under N° 2022-28 in the APHM register.

## Author contributions

LH wrote the manuscript, performed the experiments, and analyzed the data. NS, CH, PA and PB performed medical examinations, collected and analyzed data. NC performed medical examinations and helped draft the manuscript. J-MR reviewed the manuscript. SB designed the study, drafted, and revised the manuscript. All authors read and approved the final version of the manuscript.
